# Permeation Enhancer in Microemulsions and Microemulsion-Based Gels: A Comparison of Diethylene Glycol Monoethyl Ether and Oleyl Alcohol

**DOI:** 10.3390/gels11010041

**Published:** 2025-01-05

**Authors:** Sujata Pandey, Gabriella Baki

**Affiliations:** Department of Pharmacy Practice, College of Pharmacy and Pharmaceutical Sciences, The University of Toledo, 3000 Arlington Ave, Toledo, OH 43614, USA; sujata.pandey@rockets.utoledo.edu

**Keywords:** ibuprofen, microemulsion design, ternary phase diagrams, permeation enhancers, permeation

## Abstract

Microemulsions have been commonly used with various permeation enhancers to improve permeability through the skin. The purpose of this study was to compare the release and permeation ability of two commonly used permeation enhancers—diethylene glycol monoethyl ether (DGME) and oleyl alcohol—by the changes in oil composition, the addition of a gelling agent, and water content using ibuprofen as a model drug. Four microemulsions were formulated, selection was based on ternary phase diagrams, and physicochemical properties were evaluated. The release and permeation of the microemulsion formulations were performed in vitro by Franz cell studies on a regenerated cellulose membrane and a Strat-M^®^ membrane, respectively, and the amount of ibuprofen permeated and released was analyzed by high-performance liquid chromatography (HPLC). All four microemulsions were compatible with the skin pH, and the average pH ranged from 4.9 to 5.6. The average droplet size of the microemulsions ranged from 119.8 to 153.3 nm. Drug release was significantly the highest from the gel-based microemulsions (59% and 64%, *p* < 0.05). However, there was a fourfold difference in drug permeation from these gels—a significantly higher permeation from the microemulsion-gel containing oleic acid and oleyl alcohol compared to the DGME formulation. These results indicated that the microemulsion-gel with oleyl alcohol as the permeation enhancer could be a preferable formulation approach for the topical administration of ibuprofen. These results highlight the need for optimization of the microemulsion formulation to confirm the permeation-enhancing effects of chosen permeation enhancers despite being a well-known permeation enhancer.

## 1. Introduction

The skin is a selectively permeable membrane in which the stratum corneum (SC) layer provides the barrier function [1]. The entry of molecules through the skin is controlled by the stratum corneum (SC) allowing selective passage of the molecules with the molecular weight usually below 500 Daltons and lipophilicity with a log P_octanol/water_ partition coefficient ranging from 1 to 3 through the skin [2]. Other parameters influencing permeability of the skin are solubility, hydrogen bonding, and melting point [3,4,5]. Various approaches, including the utilization of physical and chemical techniques, have been performed to reduce the limitations in permeation provided by the SC and enhance dermal drug delivery. Examples of physical approaches include iontophoresis [6], microneedles [7], and sonophoresis [8,9].

A commonly used chemical approach is the use of microemulsion formulations with various permeation enhancers. Microemulsions are single-phase, transparent dispersion systems of water, oil, and surfactant, mostly in combination with fixed ratios of co-surfactant(s). Microemulsions are thermodynamically stable liquid systems compared to other emulsion systems [10]. The formation of microemulsions largely depends on the properties of the constituents used and the ratios of the oil–surfactant–water constituents. Thus, the microemulsion formation usually falls in limited concentration ranges, and the region of microemulsion formation is generally represented in pseudo-ternary phase diagrams. The pseudo-ternary phase diagrams show the regions of microemulsion formation as ratios of water, oil, and a predetermined combination of surfactant and co-surfactant ratios. The internal structure of a microemulsion affects the diffusivity of the oil and water phases [11] and thus the diffusion of a drug from the phases. An increase in the permeation capacity of oil components, surfactants, and penetration enhancers can be achieved by the following mechanisms: (1) disruptions of the lipid components of the SC layer, or (2) increasing the partition coefficient of the drug in the skin compared to the vehicle, i.e., increased solubility in the skin. [12].

Various approaches for dermal delivery of ibuprofen include microemulsions [13], microemulsions-based gels [14], hydrogel-based microemulsions systems [15], and emulgels [16]. The increment in drug loading capacity due to the amphiphilic interface and an area for enhancement of drug solubility offers advantage to microemulsions [12,17,18,19]. The reduction in droplet size and hence the increased surface area–volume ratio can lead to increased drug permeation from microemulsions. Additionally, optimization of the surfactant–co-surfactant ratio (S/Co-S) [17,20,21] is a key factor to be considered in microemulsion development.

The composition of the oil phase influences parameters including drug release, drug solubility in the microemulsion, and skin permeation [22]. The type of gelling agent has shown significant differences in the permeation, where xanthan gum was a suitable gelling agent compared to carbomer 940 in the preparation of ibuprofen microemulsion, showing higher stability [23]. However, carbomer gel showed higher permeation compared to xanthan gel in a tretinoin microemulsion [24]. The permeation effect of oleic acid was significantly improved with a decreased amount of oleic acid, a well-known permeation enhancer [25].

In this study, we compared the release and permeation effects of two commonly used permeation enhancers, DGME and oleyl alcohol, with and without the presence of a gelling agent (carbomer 940). While carbomer 940 has been studied in combination with different permeation enhancers in ibuprofen formulations, the specific comparison of DGME + carbomer 940 with oleyl alcohol + carbomer 940 has not been previously reported. This is an important distinction, as both DGME and oleyl alcohol are widely used permeation enhancers in commercial formulations. Although the properties of these enhancers have been individually reported, the data were obtained under varying conditions and therefore the comparisons may not accurately reflect the overall understanding of the permeation ability. The investigation into the effects of gelling agents on the release and permeation of microemulsions containing oleyl alcohol and DGME is valuable because their effect can vary depending on the permeation enhancers, and the specific composition of the formulation. By systematically evaluating the interplay between these formulation components, we provide novel insights that could guide the rational design of ibuprofen delivery systems. Although the focus was on comparing microemulsions and microemulsion-based gels using ibuprofen, our findings could be beneficial for other topical drugs, including topical NSAIDs, in formulation optimization to facilitate drug permeation through the skin barrier.

## 2. Results and Discussion

### 2.1. Pseudo-Ternary Phase Diagrams

Ternary phase diagrams are essential tools in the development of microemulsion systems, providing a visual representation of the phase behavior of mixtures containing oil, water, and surfactant/cosurfactant. These diagrams are used to identify the specific ratios of surfactant, cosurfactant, oil, and water that result in the formation of a stable and transparent microemulsion region. In this study, ternary phase diagrams were utilized for the identification of the microemulsion region and the conditions under which microemulsions could be consistently formulated. This approach provided an understanding of the role of each component and their interactions, which are critical for designing effective and stable microemulsion-based systems.

**Figure 1:**
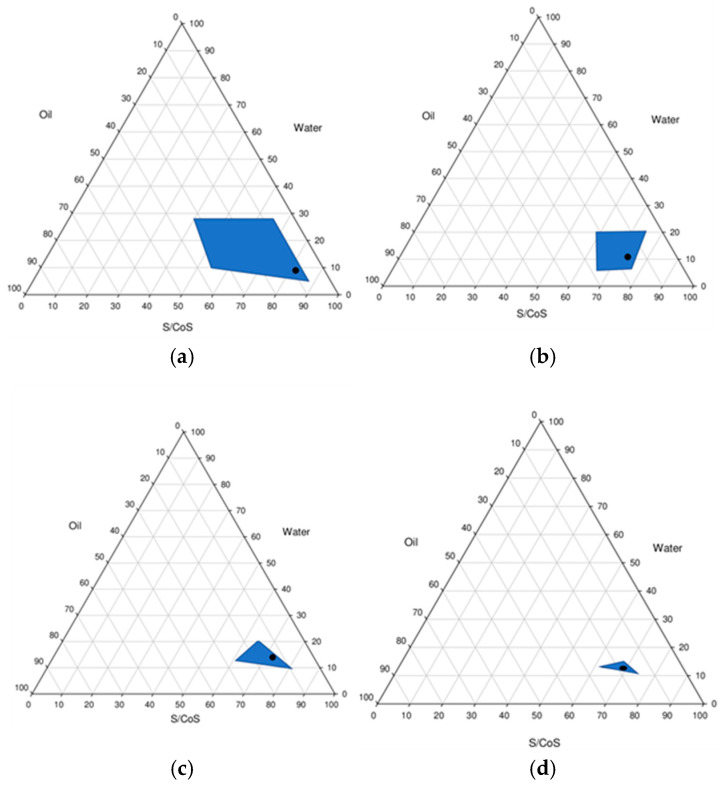
Ternary phase diagrams of (**a**) system with Polysorbate 80/oleyl alcohol (3:1), (**b**) system with Polysorbate 80/oleyl alcohol (2:1), (**c**) system with Polysorbate 80/DGME (3:1), (**d**) system with Polysorbate 80/DGME (2:1).

The region of microemulsion formation was larger with the systems of Polysorbate 80/oleyl alcohol (2:1/3:1) compared to the systems of Polysorbate 80/DGME (2:1/3:1) (Figure 1). The ratio of surfactant and co-surfactant in the microemulsions formulation was chosen (3:1) based on the larger area of microemulsion formation. The type and ratio of surfactant and co-surfactant likely resulted in differences in microemulsion formation.

### 2.2. Physicochemical Characterization

The physicochemical characterization of all four formulations is presented in Table 1. The average pH of the formulations ranged from 4.9 to 5.6, showing compatibility with the skin’s normal pH range of 4.5–5.5 [26]. The color and appearance did not change in any of the microemulsions at 25 °C during the 6-month period. F2 and F4 became less viscous based on visual observation at 40 °C during the 6-month testing period. The drug content of each formulation was analyzed at two different time points—on Day 1 and 6 months after formulation. The mean zeta potential of the formulations ranged from −0.0222 to −0.0281 mV. These values, being close to zero, indicate the absence of charged particles in the system, confirming the presence of nonionic surfactants in the formulation.

The mean droplet size of the microemulsions ranged from 119.8 to 153.3 nm, which falls in the normal range, i.e., 10–300 nm [27]. The small particle size increases the interfacial area, allowing a rapid release of the drug into the external phase [28]. The components of the microemulsions can modify the droplet size of the microemulsions. The increase or decrease in the droplet size can be altered by the volume of the oil, ratio of surfactant/co-surfactant, the structure of the polysorbates, and the total content of surfactant and co-surfactant [29].

### 2.3. Differential Scanning Calorimetry

**Figure 2:**
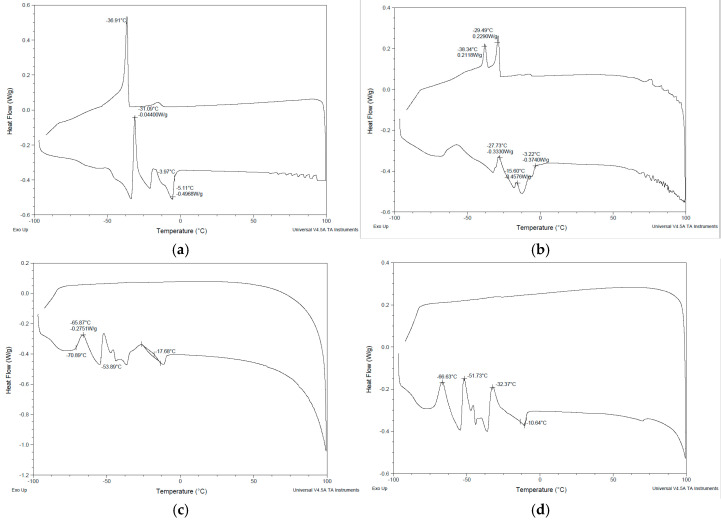
DSC thermogram of microemulsions: (**a**) F1, (**b**) F2, (**c**), F3, (**d**) F4.

Differential scanning calorimetry (DSC) is a valuable analytical technique for characterizing microstructure and analyzing the thermal behavior of microemulsion formulations. The DSC analysis provided information on the endothermic and exothermic peaks (Figure 2), which helped understand the state of the drug in the formulation and indicated the state of water; i.e., free or bound water in dispersed systems.

The solubilization of ibuprofen in the microemulsions could have led to the absence of an endothermic peak (melting peak) of ibuprofen in our study [30]. The absence of a melting peak suggests that ibuprofen is fully solubilized within the microemulsion system. In this state, the drug is no longer in its crystalline form but exists in an amorphous or molecularly dispersed form, typically dissolved in the oil phase or at the oil–water interface.

The state of water, when compared to any surface in a dispersed system, can be distinguished as ‘bulk’ (free) and ‘bound’ (interfacial) water. The physicochemical properties of bulk water are similar to that of pure water; i.e., bulk water has a freezing point/melting point nearly the same as pure water and has a similar heat of fusion [31]. In F1, an endothermic peak was seen at −3.97 °C representing the melting of bulk water (weakly bound to oleyl alcohol and Tween 80). Similarly, in F2, an endothermic peak was observed at −3.22 °C, which is nearly the same as pure water, indicating the melting of bulk (free) water. In F3 and F4, endothermic peaks appeared at −17.68 °C and −10.64 °C, respectively, which indicates bound (interfacial) water. The surfactant–water interactions have been studied by DSC measurements with reliable results [32,33,34]. Studies have reported that upon increasing the water content in a microemulsion, interactions of the surfactant and the water occurred, and bulk (free) water formed after the microemulsion was fully hydrated [34].

### 2.4. Permeation and Release Studies

#### 2.4.1. Drug Permeation and Release Studies

**Figure 3:**
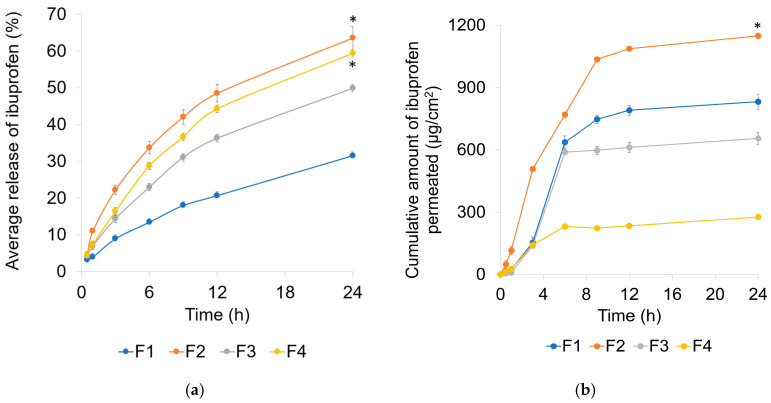
(**a**) In vitro release and (**b**) in vitro permeation of ibuprofen from the microemulsions.

Drug release was significantly higher from F2 and F4 than from F3 and F1 (*p* < 0.05, Figure 3a). The permeation was statistically significantly (*p* < 0.05) higher from F2 than from F1, F3, and F4 (Figure 3b). F2 consisted of oleic acid and oleyl alcohol with Polysorbate 80 as a surfactant. A research group found that the use of fatty acids with propylene glycol significantly increased the permeation of acyclovir [33]. It is possible that the incorporation of oleic acid with oleyl alcohol in the present study also aided in the permeation of ibuprofen through the Strat-M^®^ membrane. The combination of oleic acid and oleyl alcohol could have acted synergistically to increase the fluidity of the lipids on the surface of the membrane. The lipid fluidization by oleic acid in superficial stratum corneum layers has been reported in previous studies [34,35]. It is important to understand the interactions of these two enhancers with the stratum corneum and skin barrier components. The initial fluidization of the stratum corneum by the enhancers is essential for increasing the permeation; however, the recovery from skin barrier disruption is crucial for topical products [36]. The cumulative amount of ibuprofen permeated by 24 h was significantly higher from F2 (*p* < 0.05) than from the other formulations. Flux of F2 was the highest, statistically higher than F4 (*p* < 0.05); however, the difference between F1–F3 was not significant statistically; the difference between F2/F4 and F1/F3 was statistically significant (*p* < 0.05) (Table 2).

The lower permeation observed in the DGME formulations can likely be due to the high affinity of DGME for water [37]. This strong affinity may have caused DGME to interact with the aqueous regions of the membrane, consequently binding ibuprofen within these regions and limiting its permeation. This explanation is supported by the DSC thermograms of formulations F3 and F4, which revealed endothermic peaks at −17.68 °C and −10.64 °C, respectively. These peaks indicate the presence of bound (interfacial) water in the formulations, consistent with the hypothesis that DGME’s interaction with water contributed to the reduced permeation of ibuprofen. Studies suggest that DGME (which is present in F3 and F4) has a high preference for water [38,39,40]; therefore, it is possible that DGME was bound with water, which resulted in the absence of the melting peak of DGME in the thermograms of F3 and F4.

The microemulsions with DGME as a permeation enhancer (F3 and F4) showed a lower permeation compared to other microemulsions. It should be noted that F1 and F2 had a lower water content compared to F3 and F4. This could suggest that although F3 and F4 had a higher amount of water in the formulation than the other two formulations, DGME requires an even greater amount of water to increase the permeation compared to formulations richer in oil. In a study [41], it was found that addition of water in the formulations containing DGME increased the permeation rate. These findings were explained as the water content in the formulation increased, a greater quantity of ibuprofen moved out of the solution, producing greater thermodynamic activity, and thus resulting in increased permeability. The increased water content in the formulation decreases the solubility of ibuprofen in the vehicle, which acts as a driving force, potentially raising the thermodynamic activity of ibuprofen in the formulation and thus to increased skin permeation [42].

In contrast to these findings, the high affinity of DGME to water [43] could reduce the permeation ability of DGME itself, explaining the finding of this study. DGME binds on the aqueous regions of the SC layers in the absence or low amount of water in the formulation and therefore limiting the mobilization [39]. The presence of both carbomer and DGME in F4 resulted in a further reduction in permeation capacity, possibly due to carbomer’s ability to absorb water readily when in contact with water [44]. Upon absorption of water in the formulation by carbomer, DGME most likely became trapped in the carbomer gel due to its high affinity for water, which lowered DGME’s permeation-enhancing capacity and hence the low permeation. Additionally, the low permeation of ibuprofen from the F3 and F4 could be due to the high solubility of ibuprofen in DGME [16]. The high solubilization capacity of drug in solvents is likely to favor the drug staying in the formulation, limiting the diffusivity of the drug into the skin in the absence of a driving force [16,41,45]. In this study, microemulsions were incorporated into gel matrices to enhance their solubilization and permeation-enhancing properties, while the gel base provided structural stability and application convenience for topical delivery. By investigating the effects of composition changes, we demonstrated how these systems can be optimized for effective drug release and skin permeation.

However, spreadability and texture analysis of the microemulsion based gels (F2 and F4) was not evaluated in this study, which limits the full understanding of the impact of the gelling agent on the spreadability and, consequently, the viscosity of formulation. These parameters are important for characterizing the mechanical properties of microemulsion-based gels. The gelling agent in F4 may have impacted the spreadability and, consequently, the viscosity of the formulation.

Owing to the lipophilicity of the ibuprofen, it was incorporated into the oil phase. Scanning electron microscopic images could have further confirmed the encapsulation efficiency of the microemulsions and microemulsions gels by visualizing the microemulsions loaded with ibuprofen. The microemulsion-based gel with oleyl alcohol and oleic acid showed higher permeation in this study. The effect of oleic acid and oleyl alcohol was compared individually in ex vivo human skin in a diclofenac formulation [36], where FTIR spectra results showed a higher content of the oleic acid in the stratum corneum long after removal of the exposure compared to oleyl alcohol. The major outcomes of this study, regarding the formulation, were the release and permeation profiles in addition to the droplet size and stability parameters. Based on the increased permeation of ibuprofen, oleic acid and oleyl alcohol gel is a better formulation; however, the properties such as recovery from the barrier skin is equally important to consider.

#### 2.4.2. Drug-Release Kinetics

The drug-release percentage from the formulations was higher than the amount permeated. This difference is due to the membranes used in the studies; the membrane used in the release studies does not restrict drug permeation, while the Strat-M^®^ membrane is designed to simulate the barrier properties of skin. The type of membrane and membrane properties are known to affect the release of ibuprofen from the formulation. A study [46] demonstrated the regenerated cellulose membrane as a ‘low-flux’ implying contribution to the relatively lower release in our study. Drug-release kinetics indicated that the drug release was best described by the Higuchi model with highest linearity for all the formulations (Table 3), but a close relationship was also observed with the Korsmeyer–Peppas model, commonly used to describe drug-release kinetics from polymeric systems. The plot of zero-order kinetics indicates lower linearity compared with other kinetic models. These data suggest that the release mechanism is primarily diffusion controlled. The drug release from lipophilic ointments is known to follow the Higuchi model, [47,48] which was similar to our findings. It is important to note that the highest release was obtained from F2, which was about 63%. Several factors could contribute to the remaining drug unreleased, including the use of regenerated cellulose membrane used in our study as described above. For lipophilic drugs like ibuprofen, the hydrophilic nature of the regenerated cellulose membrane might further impede diffusion.

## 3. Conclusions

This study compared the release and permeation capabilities of two commonly used permeation enhancers, DGME and oleyl alcohol, in microemulsion and microemulsion-based gel formulations using ibuprofen as a model drug. The results demonstrated that the inclusion of a gelling agent and the choice of a permeation enhancer significantly influenced drug release and permeation. Gel-based microemulsions, particularly those containing oleyl alcohol, exhibited the highest drug release and significantly higher permeation compared to formulations with DGME.

While DGME exhibited lower permeation potential, likely due to its strong water affinity and interaction with aqueous regions of the membrane, oleyl alcohol emerged as the preferable permeation enhancer for topical ibuprofen delivery. These findings emphasize the need for optimization of microemulsion formulations to maximize and confirm the benefits of selected permeation enhancers. However, in vivo studies must be performed to measure the correlation of the in vitro design with the in vivo aspects.

## 4. Materials and Methods

### 4.1. Materials

Ibuprofen 25 received from BASF (Ludwigshafen, Germany) was used. Triethanolamine and Carbomer 940 were purchased from Making Cosmetics (Snoqualmie, WA, USA). Oleyl alcohol and oleic acid were provided by Croda (Newark, NJ, USA), Tween^®^ 80 (Polysorbate 80) was purchased from Fisher Scientific (Hampton, NJ, USA) and Transcutol^®^ (diethylene glycol monoethyl ether) was received from Gattefosse (Lyon, France). Other chemicals used were of analytical grade, which were used without additional purification. The deionized water used was from the Health Science Campus of the University of Toledo.

### 4.2. Methods

#### 4.2.1. Pseudo-Ternary Phase Diagrams

Oleic acid was used as the oil phase and Polysorbate 80 was the surfactant based on previous literature [49,50]. Oleyl alcohol and DGME were used as co-surfactants, and deionized water was used as the aqueous phase. A previous study from our lab [16] indicated that oleyl alcohol was a good solvent for ibuprofen with the solubility of 92 mg/mL (at 21 °C). The solubility of ibuprofen in oleic acid and oleyl alcohol predicted by Formulating for Efficacy^TM^ based on Hansen Solubility Parameters was similar; i.e., SolV of 47.6 for oleyl alcohol and 47.3 for oleic acid. These values indicated that oleic acid would be a good candidate for oil phase in the formulations. DGME was a suitable water-soluble skin penetration enhancer for ibuprofen in our previous study [16]. The area of microemulsion existence was determined by mixing the components in different ratios. Oil phase and blends of specific surfactants/co-surfactants and water were mixed carefully in the following ratios: 1:9, 2:8, 3:7, 4:6, 5:5, 6:4, 7:3, 8:2, and 9:1. The surfactants and co-surfactants were mixed in the ratios of 1:1, 2:1, 3:1, and 4:1 on a magnetic stir plate at 250 rpm for 5 min. The surfactant and co-surfactant were added to the oil phase, and then water was added dropwise to the resulting mixture based on the ratios of pseudo-ternary phase diagrams. The areas of microemulsion formation were plotted using software Sigma Plot^®^ software version 14 (Systat Software Inc., San Jose, CA, USA).

#### 4.2.2. Preparation of Ibuprofen-Loaded Microemulsions

The ratio of each component of the microemulsion was selected, which was based on the pseudo-ternary phase diagrams. A total of four microemulsions were formulated (Table 4), two of which (i.e., F2 and F4) contained a gelling agent, namely carbomer 940, and two did not contain a gelling agent (F1 and F3). The oil was added to the mixture of surfactant/co-surfactant. Due to the lipophilic nature of the drug, ibuprofen (5% *w*/*w*) was incorporated into the oil phase after combining the surfactant and co-surfactant blends. In the last step, the desired amount of water was added in a dropwise manner to the mixture. In the case of F2 and F4, 2% carbomer gel, prepared separately, was added to the resulting mixture slowly with continuous stirring using a magnetic stirrer at 250 rpm for 5 min. An accurately pre-measured amount of carbomer was added to deionized water, which was mixed with an overhead stirrer at 950 rpm for 15 min. The pH of the carbomer gel was adjusted to pH 6.0 with triethanolamine.

#### 4.2.3. Analysis of Ibuprofen

Ibuprofen was analyzed by High-Performance Liquid Chromatography (HPLC) (Waters Alliance e2695 separations module, Milford, MA, USA) coupled with Waters 2489 UV/Visible detector. The HPLC method used in our previous study [16] was used for this research. C18 column, 4 µm, 150 × 4.6 mm, Accucore XL was utilized in the study. The mobile phase was acetonitrile (ACN) and buffer (pH 2.5) mixed at a 60:40 (*v*/*v*) ratio. The buffer was a mixture of orthophosphoric acid: triethylamine: HPLC grade water (0.5 mL: 1 mL:1000 mL). The flow rate was 1 mL/min, and the injection volume was 10 µL. The detection wavelength of ibuprofen was 220 nm.

#### 4.2.4. Physicochemical Characteristics

The pH of the four microemulsions was determined by a pH meter (Mettler Toledo, Seven Compact, Columbus, OH, USA). Each microemulsion was tested in triplicates.

Each microemulsion was precisely weighed out and dissolved in acetonitrile (ACN). Upon complete dissolution, the solutions were filtered (EMD Millipore membrane filter) and diluted as required in ACN. Drug content analysis was performed initially 24 h after formulation and 6 months later using the above-described HPLC method. A stock solution of ibuprofen at a strength of 1 mg/mL was prepared in acetonitrile and calibration standards ranging from 0.195 µg/mL to 100 µg/mL were prepared from the stock solution. Calibration standards were run (n = 3) and the average peak area was obtained. A calibration curve was made by plotting the average peak area against the concentration of ibuprofen (µg/mL).

Stability of the microemulsions was tested for 6 months at two different temperatures (25 °C and 40 °C). The formulations were placed in centrifuge tubes and kept in stability chambers. The formulations were observed monthly for any signs of physical instability.

Zeta potential was obtained using the electrophoretic light scattering (ELS) technique. A dilution with water was performed to prepare each microemulsion at a concentration of 2% *w*/*w*, which was then further diluted 100 times. The diluted preparation was then loaded into a folded capillary cell, which has conductive points to receive electric charge. Each sample was tested four times. 

The mean droplet size was obtained using the differential light scattering (DLS) technique. Each microemulsion was analyzed in triplicates after appropriate dilution.

The microemulsion microstructure was evaluated using the Q20 Differential Scanning Calorimetry (DSC) instrument (TA Instruments, San Diego, CA, USA) and TA Instruments Universal Analysis 2000 software to generate and label different points in the thermogram. Microemulsion samples (6–9 mg) were accurately weighed in an aluminum hermetic sample pan, sealed with the lid, and run using the following steps: equilibration at −90 °C, heating at the rate of 5 °C/min to 100 °C, and cooling at the rate of 5 °C/min to −90 °C. The DSC running conditions for the components were based on the melting points of the individual components. Oleic acid, oleyl alcohol, and the mixture of these were tested using the following steps: equilibration at −25 °C and ramped at the rate of 5 °C/min to 50 °C. The DSC test of Polysorbate 80 was as follows: equilibration at −25 °C, ramped at 5 °C/min to −50 °C. DGME was equilibrated at −90 °C, and ramped at a rate of 5 °C/min to 25 °C. The DSC curves obtained from the individual components were compared with the DSC curves obtained from the different microemulsions.

#### 4.2.5. Drug Release and Permeation Studies

The release and permeation studies of F1–F4 microemulsions were performed in vitro using Franz diffusion cells with an orifice diameter of 15 mm and receptor chamber of 12 mL volume. A previously developed method from our study [16] was used for the release and permeation studies. The in vitro release studies were performed in Spectra/Por 2 dialysis membrane (12–14 kDa molecular weight). The membranes were soaked in phosphate-buffered saline (PBS) for 8 h before the study for hydration. Strat-M^®^ synthetic membranes were used for permeation studies. The receptor medium was PBS, pH 7.4, which was stirred during the experiment using a magnetic stir bar at 300 rpm.

Pre-weighed amounts of the microemulsions were uniformly spread on the membranes inside the donor chambers. To prevent evaporation, each Franz cell’s orifice and donor chamber were covered with parafilm. The release (n = 3) and permeation (n = 4) were studied in vitro for 24 h. Samples (500 μL) were taken from the receptor chamber at 0.5, 1, 3, 6, 9, 12, and 24 h.

#### 4.2.6. Statistical Analysis

Flux (J, µg/cm^2^/h) was calculated from the slope of the linear portion of the permeation curve. The apparent permeability coefficient (K_p_) was calculated by dividing the flux (J) with the initial concentration of ibuprofen in the donor chamber (C_donor_). One-way analysis of variance (ANOVA) with a Tukey post hoc analysis (SPSS software, version 25) was used to statistically analyze the release and permeation parameters. A *p* value < 0.05 was considered statistically significant.

## Figures and Tables

**Table 1:** Physicochemical characteristics of formulations.

Formulation	Drug Content (%)		Droplet Size	pH	Zeta Potential
	Day 1	Month 6	(nm)		(mV)
F1	105.0 ± 0.2	104.3 ± 0.7	153.3 ± 0.0	4.91 ± 0.02	−0.025 ± 0.20
F2	102.9 ± 0.8	88.7 ± 2.8	151.6 ± 0.0	5.56 ± 0.04	−0.022 ± 2.34
F3	106.4 ± 0.3	87.7 ± 0.0	119.8 ± 0.2	5.15 ± 0.04	−0.024 ± 0.78
F4	97.5 ± 0.9	86.8 ± 1.4	130.7 ± 0.4	4.91 ± 0.05	−0.024 ± 6.24

**Table 2:** Flux (J), permeability coefficient (K_p_), and lag time (t_lag_) of the formulated microemulsions in vitro.

Formulation	J (μg/cm^2^/h)	K_p_ × 10^−3^ (cm/h)	t_lag_ (h)
F1	124.5 ± 3.9	2.5 ± 0.007	1.2 ± 0.1
F2	127.4 ± 3.8	2.5 ± 0.007	0.3 ± 0.1
F3	119.7 ± 2.1	2.4 ± 0.004	1.3 ± 0.0
F4	40.1 ± 1.9	0.8 ± 0.004	0.2 ± 0.0

**Table 3:** Release kinetics model from microemulsion formulations.

Formulation	Zero Order	Higuchi Model	Korsmeyer–Peppas Model
Regression Coefficient			
F1	0.961	0.996	0.993
F2	0.885	0.987	0.968
F3	0.927	0.996	0.996
F4	0.915	0.994	0.993

**Table 4:** Composition of the microemulsions (%*w*/*w*).

Ingredient	F1	F2	F3	F4
Oleic acid	9.5	7.5	9.5	7.5
Polysorbate 80	57.0	45.0	49.8	39.4
Oleyl alcohol	19.0	15.0	-	-
Diethylene glycol monoethyl ether	-	-	16.7	13.1
2% Carbomer gel	-	20.0	-	20.0
Ibuprofen	5.0	5.0	5.0	5.0
Water	9.5	7.5	19.0	15.0

## Data Availability

The raw data supporting the conclusions of this article will be made available by the authors on request.
